# Supplementary material for: Anti-Cancer Effect of Chlorophyllin-Assisted Photodynamic Therapy to Induce Apoptosis through Oxidative Stress on Human Cervical Cancer
Source: Int J Mol Sci. 2023 Jul 17;24(14):11565. doi: 10.3390/ijms241411565 (PMC10380873; doi:10.3390/ijms241411565)
Supplement: Supplementary file 1 [file ijms-24-11565-s001.zip › Supplementary data_Figure S1.pdf]

Supplemental Materials to the paper

Anti-Cancer Effect of Chlorophyllin-Assisted Photodynamic Therapy to Induce Apoptosis through Oxidative Stress on Human Cervical Cancer

Seong-Yeong Heo [1,†], Yeachan Lee [2,3,†], Tae-Hee Kim [2,3], Soo-Jin Heo [1], Hwarang Shin [2,4], Jiho Lee [2,4], Myunggi Yi [3,4], Hyun Wook Kang [2,3,4,*] and Won-Kyo Jung [2,3,4,*]

1 Jeju Bio Research Center, Korea Institute of Ocean Science and Technology (KIOST), Jeju 63349, Republic of Korea
2 Marine Integrated Biomedical Technology Center, The National Key Research Institutes in Universities, Pukyong National University, Busan 48513, Republic of Korea
3 Research Center for Marine Integrated Bionics Technology, Pukyong National University, Busan 48513, Republic of Korea
4 Major of Biomedical Engineering, Division of Smart Healthcare and New-Senior Healthcare Innovation Center (BK21 Plus), Pukyong National University, Busan 48513, Republic of Korea
* Correspondence: wkang@pukyong.ac.kr; wkjung@pknu.ac.kr; Tel.: +82-51-629-5775
† These authors contributed equally to this work.

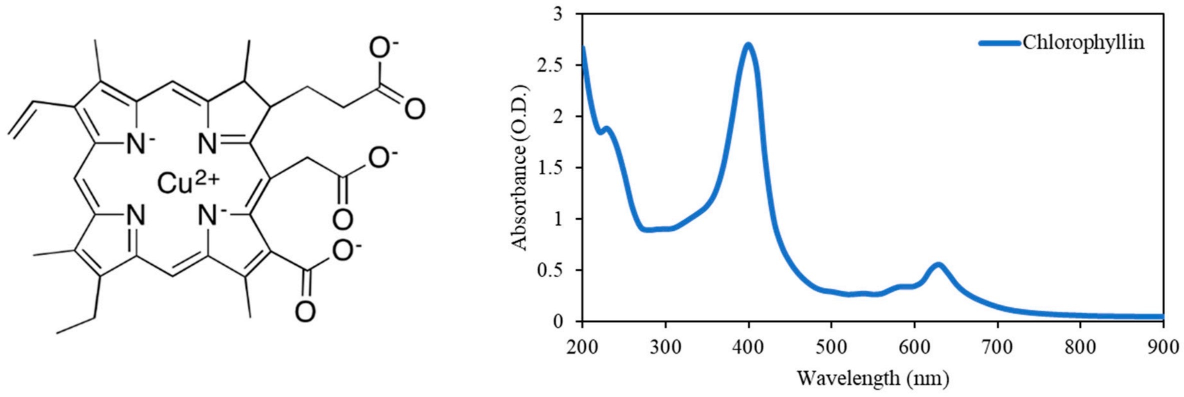

Figure S1. Chemical structure and UV-vis absorption spectra of chlorophyllin.
